# Rac and Cdc42 inhibitors reduce macrophage function in breast cancer preclinical models

**DOI:** 10.3389/fonc.2023.1152458

**Published:** 2023-06-16

**Authors:** Anamaris Torres-Sanchez, Michael Rivera-Robles, Linette Castillo-Pichardo, Magaly Martínez-Ferrer, Stephanie M. Dorta-Estremera, Suranganie Dharmawardhane

**Affiliations:** ^1^ Department of Biology, University of Puerto Rico, San Juan, Puerto Rico; ^2^ Department of Biochemistry, School of Medicine, University of Puerto Rico, San Juan, Puerto Rico; ^3^ Department of Pharmaceutical Sciences, School of Pharmacy, San Juan, Puerto Rico; ^4^ Division of Cancer Biology, University of Puerto Rico Comprehensive Cancer Center, San Juan, Puerto Rico; ^5^ Department of Microbiology, School of Medicine, University of Puerto Rico, San Juan, Puerto Rico

**Keywords:** Rac, Cdc42, MBQ-167, EHop-016, macrophage function, myeloid derived suppressor cells (MDSCs), breast cancer

## Abstract

**Background:**

Metastatic disease lacks effective treatments and remains the primary cause of mortality from epithelial cancers, especially breast cancer. The metastatic cascade involves cancer cell migration and invasion and modulation of the tumor microenvironment (TME). A viable anti-metastasis strategy is to simultaneously target the migration of cancer cells and the tumor-infiltrating immunosuppressive inflammatory cells such as activated macrophages, neutrophils, and myeloid-derived suppressor cells (MDSC). The Rho GTPases Rac and Cdc42 are ideal molecular targets that regulate both cancer cell and immune cell migration, as well as their crosstalk signaling at the TME. Therefore, we tested the hypothesis that Rac and Cdc42 inhibitors target immunosuppressive immune cells, in addition to cancer cells. Our published data demonstrate that the Vav/Rac inhibitor EHop-016 and the Rac/Cdc42 guanine nucleotide association inhibitor MBQ-167 reduce mammary tumor growth and prevent breast cancer metastasis from pre-clinical mouse models without toxic effects.

**Methods:**

The potential of Rac/Cdc42 inhibitors EHop-016 and MBQ-167 to target macrophages was tested in human and mouse macrophage cell lines via activity assays, MTT assays, wound healing, ELISA assays, and phagocytosis assays. Immunofluorescence, immunohistochemistry, and flow cytometry were used to identify myeloid cell subsets from tumors and spleens of mice following EHop-016 or MBQ-167 treatment.

**Results:**

EHop-016 and MBQ-167 inhibited Rac and Cdc42 activation, actin cytoskeletal extensions, migration, and phagocytosis without affecting macrophage cell viability. Rac/Cdc42 inhibitors also reduced tumor- infiltrating macrophages and neutrophils in tumors of mice treated with EHop-016, and macrophages and MDSCs from spleens and tumors of mice with breast cancer, including activated macrophages and monocytes, following MBQ-167 treatment. Mice with breast tumors treated with EHop-016 significantly decreased the proinflammatory cytokine Interleukin-6 (IL-6) from plasma and the TME. This was confirmed from splenocytes treated with lipopolysaccharide (LPS) where EHop-016 or MBQ-167 reduced IL-6 secretion in response to LPS.

**Conclusion:**

Rac/Cdc42 inhibition induces an antitumor environment via inhibition of both metastatic cancer cells and immunosuppressive myeloid cells in the TME.

## Introduction

Metastasis is the number one cause of death from breast cancer, with few effective clinical strategies to prevent the accelerated metastatic progression (1). During tumor initiation, growth, and metastasis, the innate and adaptive immune systems modulate anti- and pro-tumoral immune responses. Once established, tumor cells hijack the immune system to induce metastatic cancer cell migration into the circulatory system and evade immune-mediated cancer cell apoptosis (2, 3).

Cancer cell motility and invasion are essential drivers of metastasis, where the first intravasation step involves migration away from the primary tumor and extravasation into the new site of colonization using motile mechanisms (1, 3). Rac and Cdc42 are homologous Rho family GTPases that are essential for directed cell migration and promote epithelial-to-mesenchymal transition, transcription, cell proliferation, cell cycle progression, angiogenesis, apoptosis, vesicle trafficking, and cell adhesions. and thus, therapy resistance (4–6). Accordingly, deregulated expression/activity of Rac and Cdc42 is associated with increased invasion and metastasis, leading to poor patient prognosis. Even when Rac and Cdc42 are not overexpressed or mutated in cancer, they can be activated by a myriad of oncogenic cell surface receptors and guanine nucleotide exchange factors (GEFs) that exchange the GDP on inactive Rac/Cdc42 for a GTP.

In addition, immune cells such as macrophages, neutrophils, and Myeloid-Derived Suppressor Cells (MDSCs) are recruited to the tumor microenvironment (TME) to promote tumor cell invasion of the surrounding tissue, intravasation and survival in the circulation, as well as tumor cell arrest, extravasation, and persistent growth at metastatic sites, and is correlated with poor patient prognosis (7–13). Moreover, tumor-associated macrophages (TAMs) and neutrophils suppress CD8+ T cell infiltration, and thus anti-tumor immunity, to promote tumor progression and therapy resistance (14, 15). TAMs may become activated *via* Toll-like receptor (TLR) engagement leading to the secretion of cytokines and growth factors that promote an immunosuppressive environment as well as the survival of tumor cells (16, 17). Additionally, macrophages are phagocytic cells that after uptake of tumor antigens or apoptotic tumor cells have the capacity to modulate adaptive immune responses. Accordingly, therapies targeting macrophage infiltration into the tumor may provide additional treatment options for metastatic cancer patients (18).

Rac and Cdc42 also modulate the migration of macrophages and neutrophils, as well as phagocytosis, differentiation, and activation of M2-like macrophages and MDSCs, through the modulation of actin cytoskeleton structures and receptor-mediated signaling (19). Moreover, TLR signaling by invading pathogens or during cancer-associated inflammation, activate Rac1 and Cdc42 which leads to Nuclear Factor kB (NFκB) transcriptional activity and secretion of inflammatory cytokines such as interleukin-6 (IL-6) (20). Therefore, Rac is a pivotal regulator of leukocyte migration and activation relevant for immune suppression in the TME (2, 21–24).

We posit that Rac and Cdc42 orchestrate the cross-talk signaling and invasion of immune cells that migrate into the tumor and the metastatic cancer cells that leave the primary tumor. Whether the novel Rac and Cdc42 inhibitors, developed by our group, affect leukocyte migration and function, especially during cancer development, remains to be determined.

In our program to develop Rac/Cdc42 inhibitors as anti-metastatic cancer agents, we first characterized EHop-016, which inhibits Rac activation by the GEF Vav with a half maximal inhibitory concentration (IC^50^) of 1 μM and inhibits tumor growth and metastasis in mouse models (25). EHop-016 structure was improved to yield MBQ-167, which inhibits Rac/Cdc42 activation with 0.1 μM IC50. MBQ-167 induces a loss in cell polarity and cell surface actin extensions, cell cycle arrest, and apoptosis without affecting normal epithelial cells. MBQ-167 also inhibits metastatic breast cancer cell proliferation and primary tumor growth and strongly prevents metastasis (26, 27). Both EHop-016 and MBQ-167 are not toxic to rodents and MBQ-167 has an excellent safety profile in both rodents and dogs up to 1000 mg/kg BW. Moreover, EHop-016 and MBQ-167 have acceptable bioavailability in mouse plasma and tissue and MBQ-167 is available in tumors at sufficient concentrations to exert anticancer effects (28). The objective herein was to determine whether EHop-016 and MBQ-167 affect macrophage migration and function in the context of breast cancer. This study sheds light on the effects of Rac and Cdc42 inhibitors on immune response modulation within the tumor microenvironment.

## Materials and methods

### Reagents

Splenocytes were cultured in RPMI 1640 medium (from Fisher Scientific, Waltham, MA) supplemented with 10% fetal bovine serum, 50 units/mL penicillin, and 50 µg/mL streptomycin (from Fisher Scientific, Waltham, MA). Lipopolysaccharide (LPS) was obtained from Sigma-Aldrich (Burlington, MA). The following antibodies were obtained from Biolegend (San Diego, CA) and used for flow cytometry analysis: FITC anti-mouse CD11b (clone: 29F.1A12), PE-CF594 anti-mouse F4/80 (clone:BM8), PerCPCy5.5 anti-mouse CD11c (clone: N418), PE anti-mouse CD86 (clone: GL-1), BV421 anti- mouse MHC-class II (clone: M5/114.15.2). The following antibodies were obtained from Biolegend (San Diego, CA) and used for flow cytometry analysis: FITC anti-mouse CD11b (clone: 29F.1A12), PE-CF594 anti-mouse F4/80 (clone: T45-2342), BV605 anti-mouse Ly6C (clone: HK1.4), BV711 anti-mouse Ly6G (clone: 1A8) PerCPCy5.5 anti-mouse CD11c (clone: N418), PE anti-mouse CD86 (clone: GL-1), BV421 anti- mouse MHC-class II (clone: M5/114.15.2).

### Cell lines

RAW 264.7 (TIB-71), murine macrophage-like cells, and THP-1 (TIB-202) cells were obtained from ATCC (Manassas, VA). The RAW 264.7 cells were cultured with DMEM media with 1% Penicillin/Streptomycin and 10% Fetal bovine serum (Fisher Scientific, Waltham, MA), and the THP- 1 cells were cultured in RPMI media. For experiments, the THP-1 monocytic cells were differentiated into macrophages with 100 nM phorbol 12-myristate 13-acetate (PMA) for 2 days. Cell lines were authenticated by ATCC.

### Rac/Cdc42 activity assay

The GST-p21-binding domain of PAK1 conjugated to glutathione-Sepharose was used to pulldown active GTP bound Rac or Cdc42. The pulldowns (active Rac-GTP or Cdc42-GTP) and total cell lysates were separated in a 12% SDS-PAGE gel and identified by western blotting with anti-Rac specific or anti-Cdc42 antibodies (Cell Signaling, Inc., Danvers, MA). Integrated density of positive bands of active and total bands was quantified using Image J software, as per developer (NIH)’s instructions.

### MTT assay

Promega (Madison, WI) CellTiter 96^®^ Non-Radioactive Cell Proliferation Assay (MTT) was used to quantify the viability of macrophage cells treated with MBQ-167 and EHop-016 for 48 hrs. After the incubation, the MTT reagent was added to the experimental plates and left to incubate for 4 hrs at 37°C and 5% CO_2_. Results were obtained by reading the absorbance at 570 nm wavelength, and the appropriate controls used, as per manufacturer’s instructions.

### Wound-healing assay

RAW 264.7 cells were seeded in a 24-well plate until 95-100% confluency, and serum starved overnight. A wound was created in the center with a pipet tip and treated with vehicle, MBQ-167, or EHop-016, for up to 48 hrs. Images were captured using a Keyence Microscope system and a wound- healing size tool plug-in for ImageJ software (US National Institutes of Health (NIH)) was used to quantify the wound area, as per developer’s instructions. The wound area was quantified at time 0 and 48hrs using the ImageJ plugin for wound healing assays. The % wound closure was calculated using the following formula: [Area of wound at 48hrs/Area of wound at 0hrs]x100.

### Immunofluorescence microscopy

THP-1 cells were seeded 1X10^5^ cells/mL and differentiated as described, then treated with vehicle, MBQ-167, or EHop-016 for 24 hrs. Cells were fixed with 3.7% formaldehyde and permeabilized with 0.2% Triton-X 100. Rhodamine Phalloidin Reagent (ab235138, Abcam, Cambridge, UK) was used to stain F-actin. Anti p-PAK (1/2/3) Thr 423/402/421 antibody (ab62155, Abcam, Cambridge, UK) conjugated to Alexa-488 (ab150077, Abcam, Cambridge, UK) was used to stain p-PAK. The intensity of the p-PAK fluorescence from the fluorescein-tagged secondary antibody specific for the primary anti-p-PAK antibody was quantified using Image J software. The image processing guidelines provided by the developers of Image J was used to quantify fluorescence area and integrated density.

### *In vitro* culture of splenocytes

Single-cell suspensions from extracted spleens of naïve SCID mice were obtained after passing through a 70 µm cell strainer and red blood cells were lysed with ACK lysis buffer obtained from Abcam Gibco Thermo Fisher (Waltham, MA). Cells were cultured at 1X10^6^ cells/mL with or without LPS at 10 µg/mL and treated with vehicle, MBQ-167, or EHop-016. After 24 hrs, supernatants were collected for cytokine quantification by ELISA, as per manufacturer’s instructions (R&D Systems, Minneapolis, MN) and the cells were harvested for flow cytometry analysis.

### Animal protocols

As published by us, immunocompromised mice were inoculated at the mammary fat pad with a green fluorescent protein (GFP)-tagged human metastatic cancer cell lines HER2++BM or MDA-MB-231, while the immunocompetent BalB/c mice were inoculated with 4T-1 mouse breast cancer cells (27). Once the tumors reached ~100 mm^3^ in Experiment 1, nude mice with HER2++BM tumors were treated with vehicle (12.5% ethanol, 12.5% Cremophor, Sigma-Aldrich, St. Louis, MO) or 30 mg/kg BW EHop-016 intraperitoneally (IP) 3X a week for 55 days, as described in (29). Mouse tumors were extracted at necropsy, fixed in 10% buffered formaldehyde, and paraffin-embedded for immunohistochemistry and immunofluorescence, as described below.

Experiment 2 GFP- tagged HER2++BM cells were inoculated at the mammary fatpads of SCID mice. Mice were imaged in UVP iBox *In Vivo* imaging system (Analytik Jena, Jena, Germany), and fluorescence Tumor growth was analyzed by integrated density of fluorescence intensity using image J software. When the tumors were ~100 mm^3^, the mice were treated with vehicle (12.5% ethanol, 12.5% Cremophor) or 5 mg/kg BW MBQ-167 5X a week by IP for 60 days, as described in (27, 30). At the end of the study, tumors and spleens were processed for flow cytometry analysis as described below.

Experiment 3: Balb/C mice were inoculated at the mammary fatpad with GFP-4T-1 cells, when the tumors reached 100 mm^3^, the mice were treated with vehicle (0.5% methyl cellulose, 0.1% Tween 80 in PBS) or 50 mg/kg MBQ-167 per oral (P.O.) for 28 days (27). At the end of the study, tumors and spleens were processed for flow cytometry analysis, as described below.

### Immunohistochemistry and immunofluorescence of tumor tissue

Formalin fixed paraffin embedded (FFPE) tumor samples were dewaxed in xylene and rehydrated in descending concentrations of alcohol. Antigen retrieval was performed using heat and a citrate-based Antigen Unmasking Solution (1:100 dilution) (Vector Laboratories, Burlingame, Ca, USA). Endogenous peroxidase was quenched with 3% v/v H_2_O_2_. The primary antibodies used were: F4/80 (1:1000 dilution) (ab16911, Abcam, Cambridge; MA, USA) and neutrophil elastase (1:1000 dilution) (ab68672, Abcam, Cambridge; MA, USA). Immunohistochemistry was detected using Dako Envision system-HRP (DAB) (anti-rabbit) (Dako; Glostrup, Denmark) or Dako LSAB System-HRP (DAB) (anti-mouse) (Dako; Glostrup, Denmark) according to the manufacturer’s instructions. Hematoxylin was used as a counterstain.

For immunofluorescence, the secondary antibody used was Alexa-Fluor 594 (anti-rabbit) 1:2000 (Molecular Probes, Life Technologies, Carlsbad, CA, USA) and nuclei were stained with DAPI 1:5000 (Santa Cruz Biotechnology, Santa Cruz, CA, USA). To quantify F4/80 and elastase, 3 random fields were chosen per slide and the total number of positive cells was counted for 5 slides/treatment. Statistical analysis was done using the Student’s T-test at a 95% confidence interval. n = 5 representative tumors per group.

### Flow cytometry

At the end of each *in vivo* experiment, tumors and spleens were collected, and single-cell suspensions were obtained. The process for obtaining single-cell suspensions on spleens is described above. For isolation of tumor-infiltrating leukocytes (TILs), tumors were minced and digested using 0.5 mg/mL of collagenase D, followed by a Percoll gradient separation. The flow cytometry staining protocol was followed as described in (31). A cocktail of antibodies against CD11b, F4/80, Ly6C, Ly6G, CD11c, CD86, MHC-class II was used to quantify different myeloid cell populations and their activation status. Live/Dead Aqua cell marker (Thermo Fisher, Waltham MA) was used to exclude dead cells. Also, anti-CD16/CD32 antibody was used to prevent non-specific binding of antibodies to Fc receptors. Stained cells were fixed with BD Cytofix/Cytoperm (BD Bioscences, San Jose CA) and prepared for acquisition on a FACSCelesta analyzer (BD Bioscences, San Jose CA). Data were analyzed using FlowJo Software v10 (FlowJo, LLC, Ashland, OR).

### ELISA assays

Concentrations of IL-6 on cell culture supernatants and mouse plasma were quantified by using commercially available ELISA kits from R&D Systems, as per manufacturer’s instructions (Minneapolis, MN).

### Phagocytosis assays

Phagocytosis assays were conducted in RAW 264.7 cells, following treatment with EHop-016 (1-2 μM) or MBQ-167 (250-500 nM) for 6 hrs. Cells were then proceeded to phagocytize fluorescently-tagged Zymosan particles for 2.5 hrs (Abcam, Cambridge, UK). The fluorescence from the Zymosan particles was quantified at 490/520nM in a Bio-Rad fluorescence microplate reader using manufacturer’s directions for assay management, analysis software, and performance verification tools (Bio-Rad, Hercules, CA). Representative images were taken using a Keyence digital microscope, as per instructions provided with the Keyence software (Keyence, Corp, Osaka, Japan). The obtained values from the microplate reader were first subtracted to the no-cell negative control wells and the phagocytosis response calculated using: [experimental phagocytosis sample/positive phagocytosis control]x100 (Abcam, Cambridge, UK).

## Results

### Rac/Cdc42 inhibitors reduce Rac and Cdc42 activation in macrophage-like cell lines

We characterized the small molecule compound EHop-016 as a specific inhibitor of the interaction between the GEF Vav and Rac with an IC50 of 1 μM for Rac activation in breast cancer and leukemia cells (25, 32). EHop-016 inhibits Cdc42 activation and the viability of breast cancer cell lines at much higher concentrations (~10 μM) (25). The improved EHop-016 derivative MBQ-167 demonstrated 10X higher efficacy at 100 nM IC50 for inhibition of Rac1 activation and 78 nM for Cdc42 activation in breast cancer cells (26). To determine whether these inhibitors have similar effects on macrophages, we utilized the THP-1 human monocyte-like cell line differentiated into macrophages with phorbol 12- myristate-13-acetate (PMA). THP-1 cells were treated with 0 – 4000 nM EHop-016 or 0 – 500 nM MBQ-167 and a pulldown assay was performed using the Rac.GTP/Cdc42.GTP binding domain of their downstream effector p21-activating kinase (PAK). The westerns were probed with a pan Rac (1–3) antibody, but we expect the positive bands to be mostly Rac2, since hematopoietic cells, such as macrophages, predominantly express the Rac2 isoform (33). Rac and Cdc42 activation were only quantified from the adherent differentiated macrophages and not the non-adherent non-differentiated monocytes. Future studies will also ascertain if the Rac/Cdc42 inhibitors affect the undifferentiated monocytes.

**Figure 1:**
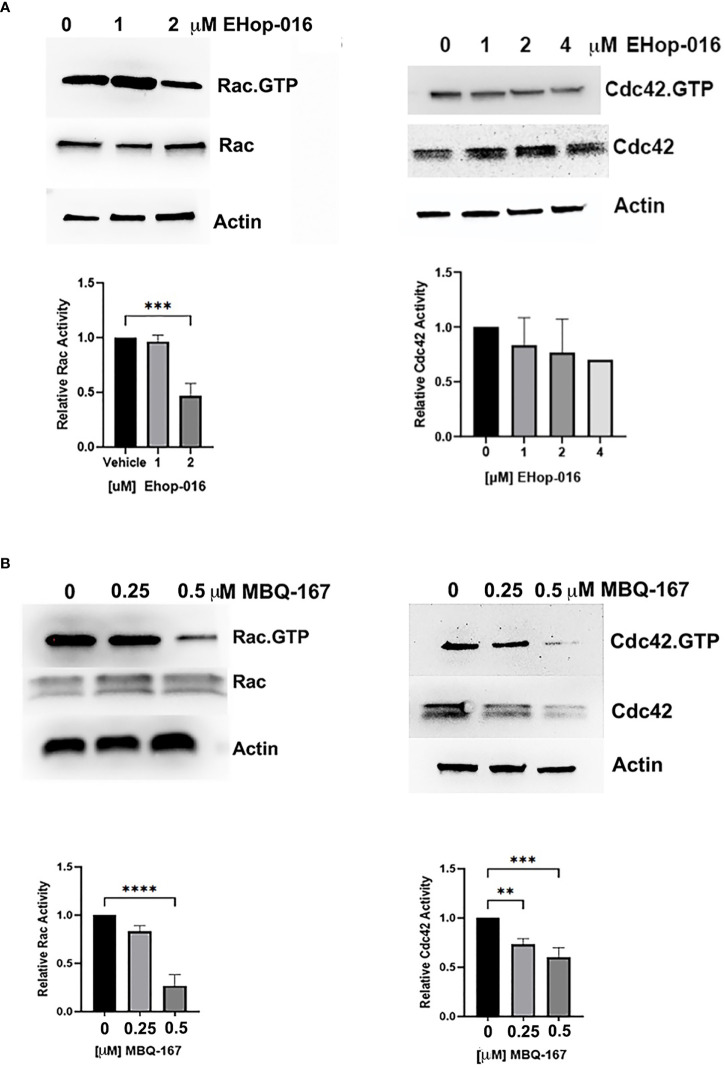
Effect of EHop-016 and MBQ-167 on macrophage Rac and Cdc42 activation. THP-1 monocytes were differentiated into macrophages, and adherent cells were treated with **(A)** EHop-016 (0 – 4000 nM) or **(B)** MBQ-167 (0-500 nM) for 24 hrs. Rac or Cdc42 activation was quantified by incubation of cell lysates with Sepharose beads containing GST-tagged p21-binding domain of PAK1. Pulldowns and total lysates were Western blotted with a pan Rac (1–3) or Cdc42 antibody. Top panel, Representative Westerns (N=3) are shown, with actin as a loading control. Bottom panel, Positive bands were quantified using ImageJ and Rac or Cdc42 activation quantified as Rac.GTP/total Rac or Cdc42.GTP/total Cdc42 relative to vehicle (1.0). N=5, **p<0.005, ***p<0.0005, ****p<0.00005.

EHop-016 reduced Rac-GTP levels by ~60% at 2 µM and by 80% at 4 µM in THP-1 human macrophages, while active or total Cdc42 remained unchanged up to 4 µM EHop-016 (**Figure 1A**). MBQ-167 reduced Rac-GTP levels by 20% at 250 nM and by 75% at 500 nM. MBQ-167 also inhibited Cdc42 activation in THP-1 macrophage-like cells by ~25% at 250 nM and by ~40% at 500 nM. At 500 nM, MBQ-167 also reduced Cdc42 expression, which may be due to the previously reported induction of anoikis by MBQ-167 at high concentrations (26) or a specific effect on Cdc42 expression or stability (**Figure 1B**). Therefore, EHop-016 and MBQ-167 inhibit Rac and Cdc42 activation in macrophage cell lines at concentrations ~5X higher than the effective concentrations for Rac and Cdc42 inhibition in breast cancer cell lines (See full length westerns in **Supplementary Figure S1**). This result can be attributed to the higher expression of Rac and Cdc42 in macrophages compared to breast cancer cells. As shown in **Supplementary Figure S2**, when equal protein was loaded from macrophage or MDA-MB-231 TNBC cell lysates, as demonstrated by equal Actin staining, a ~60% reduction in Rac expression and a 45% reduction in Cdc42 expression was observed in TNBC cells compared to THP-1 human macrophages (**Supplementary Figure S2A**). Similarly, a 60% reduction in Rac expression and an 86% reduction in Cdc42 expression in TNBC cells was observed when compared to RAW264.7 mouse macrophages (**Supplementary Figure S2B**).

### Rac/Cdc42 inhibitors do not affect macrophage cell viability at effective concentrations

**Figure 2:**
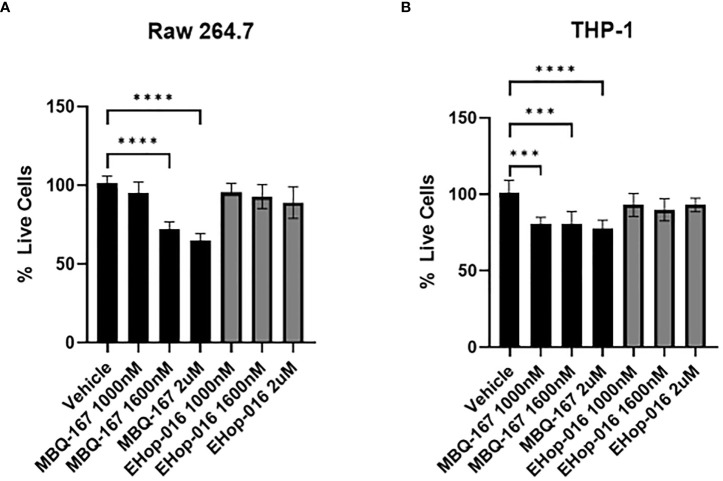
Effect of EHop-016 and MBQ-167 on macrophage viability. RAW 264.7 mouse macrophage-like cells **(A)** or THP-1 monocytes differentiated into macrophages **(B)** were treated with 0-2000 nM MBQ-167 or EHop-016 for 48hrs and the viability determined by an MTT assay (N = 5). N=5, **p<0.005, ***p<0.0005, ****p<0.00005.

To determine whether Rac/Cdc42 inhibitors affect the viability of macrophages at the therapeutic window, i.e. IC50 of 1.1 µM for EHop-016 and IC50 of 100 nM for MBQ-167 in breast cancer cells (25, 26), we determined cell viability as measured by an MTT assay on RAW264.7 and THP-1 cell lines. In RAW 264.7 cells, MBQ-167 inhibited cell viability by 20% at 1 μM and by 25% at 2 μM, while EHop-016 had no effect at similar concentrations (**Figure 2A**). A similar ~20% decrease in cell viability was also observed at >1 μM MBQ-167 in THP-1 macrophage-like cells, with no response to EHop-016 at concentrations up to 2 μM (**Figure 2B**). We found that EHop-016 at 10 μM inhibited RAW 264.7 mouse macrophage-like cell viability by 50%; however, EHop-016 at the same concentration was more potent in the 4T-1 mouse TNBC cell line (**Supplementary Figure S3**). Since the MTT assay measures metabolic activity which may be affected by cell death and/or proliferation, we quantified the frequency of dead RAW 264.7 cells by flow cytometry after 24 hrs of incubation with the inhibitors. We determined that no effect on the percentage of live cells was detected after treatment (**Supplementary Figure S4**). These data demonstrate that the Rac/Cdc42 inhibitors MBQ-167 and EHop-016 inhibit Rac activity without affecting the viability of macrophage-like cells.

### Rac/Cdc42 inhibitors reduce downstream signaling to the actin cytoskeleton and inhibit cytoskeletal extensions, migration, and phagocytosis

**Figure 3:**
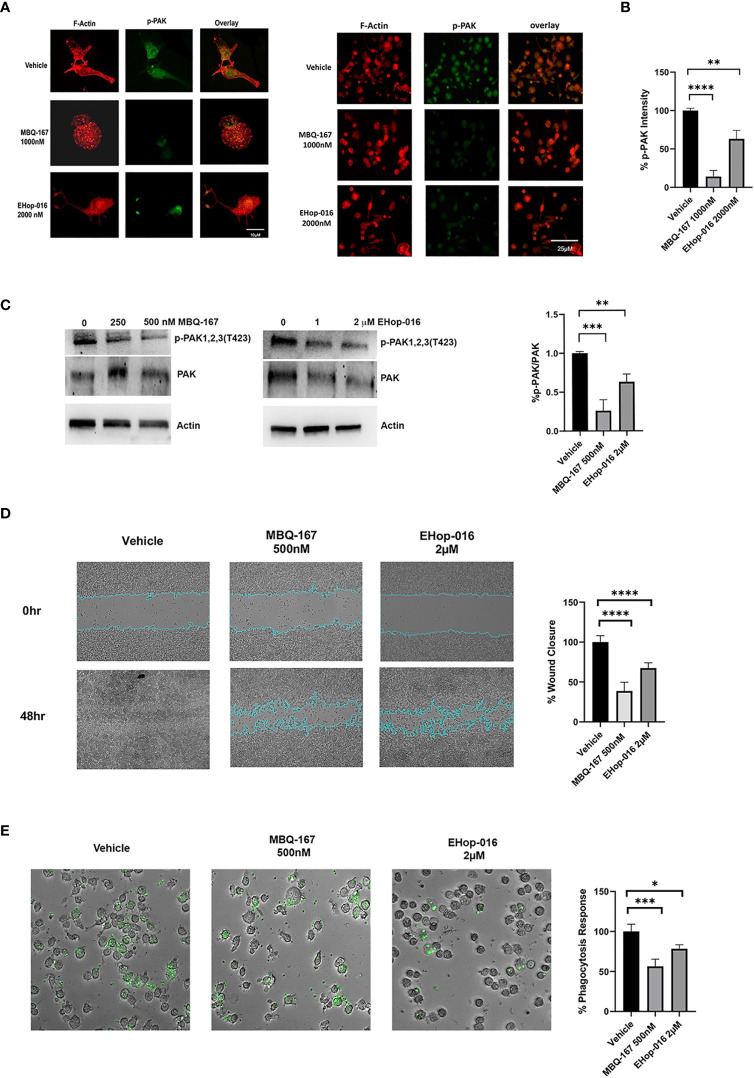
Rac/Cdc42 inhibitors reduce PAK activity, cell surface actin structures, cell migration, and phagocytosis of macrophages. **(A)** Rac/Cdc42 inhibitors reduce PAK activity and cell surface actin structures. THP-1 cells growing on coverslips were differentiated with PMA and treated with vehicle, MBQ-167 (1000 nM), or EHop-016 (2000 nM) for 24hrs. Cells were fixed and F-actin was stained with Rhodamine Phalloidin (red) and active PAK immunostained with phospho (p)-PAK Alexa 488 conjugated antibody (green). Left, representative images (400x). Right, representative images (200x). **(B)** quantification of staining intensity using image J software (N = 4 from 10 microscopic fields/biological replicate). **(C)** PAK phosphorylation in response to MBQ-167 and EHop-016. THP-1 macrophages were treated with 0, 250, or 500nM MBQ-167 or 0, 1, 2 μM EHop-016 for 24hrs and lysed. Cell lysates containing equal protein (25 μg) were Western blotted for PAK or active phospho (p-) PAK with specific antibodies to PAK 1,2,3 isoforms. Actin is shown as a loading control. Left, Representative Western blots. Right, Quantification of the integrated density of positive bands using Image J software. p-PAK/PAK relative to vehicle (100%) N=3. **(D)** Rac/Cdc42 inhibitors reduce macrophage cell migration. Raw 264.7 cells were grown to confluence and treated with vehicle, MBQ-167 (500 nM), or EHop-016 (2000 nM). A wound was created with a pipet tip and cells were allowed to migrate for 48hrs. Representative images (left) and image J quantification of wound area (right) for N=5 are shown. *p<0.05. **(E)** MBQ-167 and EHop-16 inhibit phagocytosis. RAW 264.7 cells were treated with EHop-016 (2000 nM) or MBQ-167 (500 nM) for 6hrs, then incubated with green fluorescent Zymosan particles for 2.5 hrs and imaged using a Keyence microscope system. Left, representative images (10x); right, quantification of ingested fluorescent particles. N=5. For all graphs, *p < 0.05, **p<0.005, ***p<0.0005, ****p<0.00005.

Activated (GTP bound) Rac and Cdc42 activate their common downstream effector p21-activated kinase (PAK) *via* phosphorylation to induce actin cytoskeleton reorganization into lamellipodia, filopodia, and invadopodia required for directed cell migration and invasion (34). Therefore, we wanted to determine the effect of Rac/Cdc42 inhibitors on the actin cytoskeleton on differentiated THP-1 macrophages by staining with Rhodamine phalloidin, which specifically stains F-actin filaments, and phosphorylated PAK (p-PAK, Alexa 488 fluorescence). THP-1 differentiated macrophages were treated with vehicle, EHop-016 or MBQ-167 for 24 hrs. Vehicle-treated differentiated THP-1 macrophages with PMA showed F-actin organized into cytoskeletal structures in polarized cells. p- PAK fluorescence was also observed both in the cytoskeletal extensions and in the cytosol and at the nuclear periphery. Treatment with EHop-016 at 2 µM reduced p-PAK levels and actin cytoskeletal structures compared to vehicle. F-actin was observed in a punctate distribution in the cytosol, which was more evident following 500 nM MBQ-167. MBQ-167 treatment resulted in loss of polarity in the macrophage cells, similar to the phenotype previously reported in breast cancer cell lines (26). This reduction in actin cytoskeletal structures in response to MBQ-167 was paralleled by reduced p-PAK staining intensity (**Figures 3A, B**). The reduction in p-PAK immunostaining in response to MBQ-167 or EHop-016 was also confirmed by Western blotting with antibodies to PAK or p-PAK. Results show a ~70% decrease in p-PAK levels by 250 and 500nM MBQ-167 and a 14% decrease by 1 μM and a ~30% decrease in p-PAK levels by 2 μM EHop-016 (**Figure 3C**).

Since actin cytoskeleton reorganization is critical for cellular migration, we next determined the effect of Rac/Cdc42 inhibitors on cell migration. Wound-healing assays of RAW 264.7 cells show that EHop-016 at 2 µM significantly inhibited macrophage cell migration by 38.3%, while MBQ-167 at 500 nM significantly inhibited macrophage cell migration up to 66.6% (**Figure 3D**). This data suggests that Rac/Cdc42 inhibitors impair actin cytoskeleton reorganization to impede migration of macrophages. Since phagocytosis is an essential function of macrophages, which involves Rac and Cdc42 regulated actin cytoskeletal rearrangement (35), we tested the effect of Rac/Cdc42 inhibitors on the phagocytosis of fluorescent beads. **Figure 3E** shows that 500 nM MBQ-167 reduced phagocytosis of RAW 264.7 macrophages by 50%, while 2 μM EHop-016 exerted a 75% decrease in phagocytosis.

### Rac and Cdc42 inhibitors reduce myeloid cell activation and infiltration into mammary tumors

**Figure 4:**
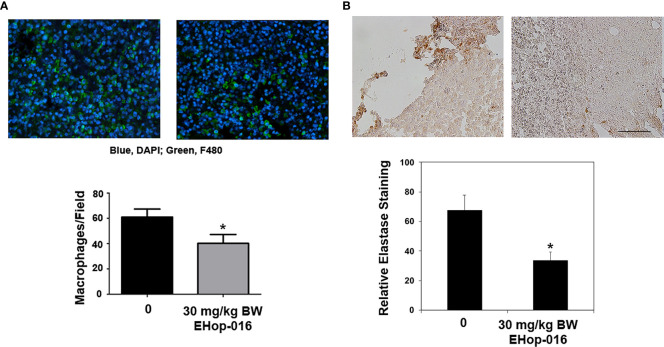
Effect of EHop-016 on leukocyte infiltration into mammary tumors. As described in (29), mammary fat pad tumors were established from GFP-HER2-BM cells in nude mice and treated with vehicle or 30 mg/kg BW EHop-016 by i.p. 3X a week for 55 days. Primary tumors were fixed and processed by immunostaining for **(A)** macrophages (F4/80) or **(B)** neutrophils (elastase) (right). Representative micrographs and quantification of positive staining is shown for N=5, *p<0.05. Scale bar 50μM.

To determine whether Rac and Cdc42 inhibitors affect leukocyte migration *in vivo*, we quantified leukocytes by immunofluorescence or flow cytometry in tumors or spleen in preclinical breast cancer mouse models. Immunocompromised athymic nude mice were implanted with GFP-HER2-BM human cancer cells in mammary fat pads and treated with vehicle or 30 mg/kg BW EHop-016 by intraperitoneal (IP) route 3 times a week for 55 days. We previously published that EHop-016 treatment resulted in ~90% reduction in tumor growth, angiogenesis, and metastasis (29). At the end of the study, GFP-fluorescent tumors (as ascertained by *in situ* fluorescence imaging) were extracted, fixed, and immunostained for macrophages (F4/80 positive). Results show reduced macrophage infiltration in tumors from EHop-016 treated mice compared to vehicle-treated mice (**Figure 4A**). We also observed decreased neutrophil infiltration in EHop-016 treated mice as quantified in immunostained tissues for elastase (**Figure 4B**).

**Figure 5:**
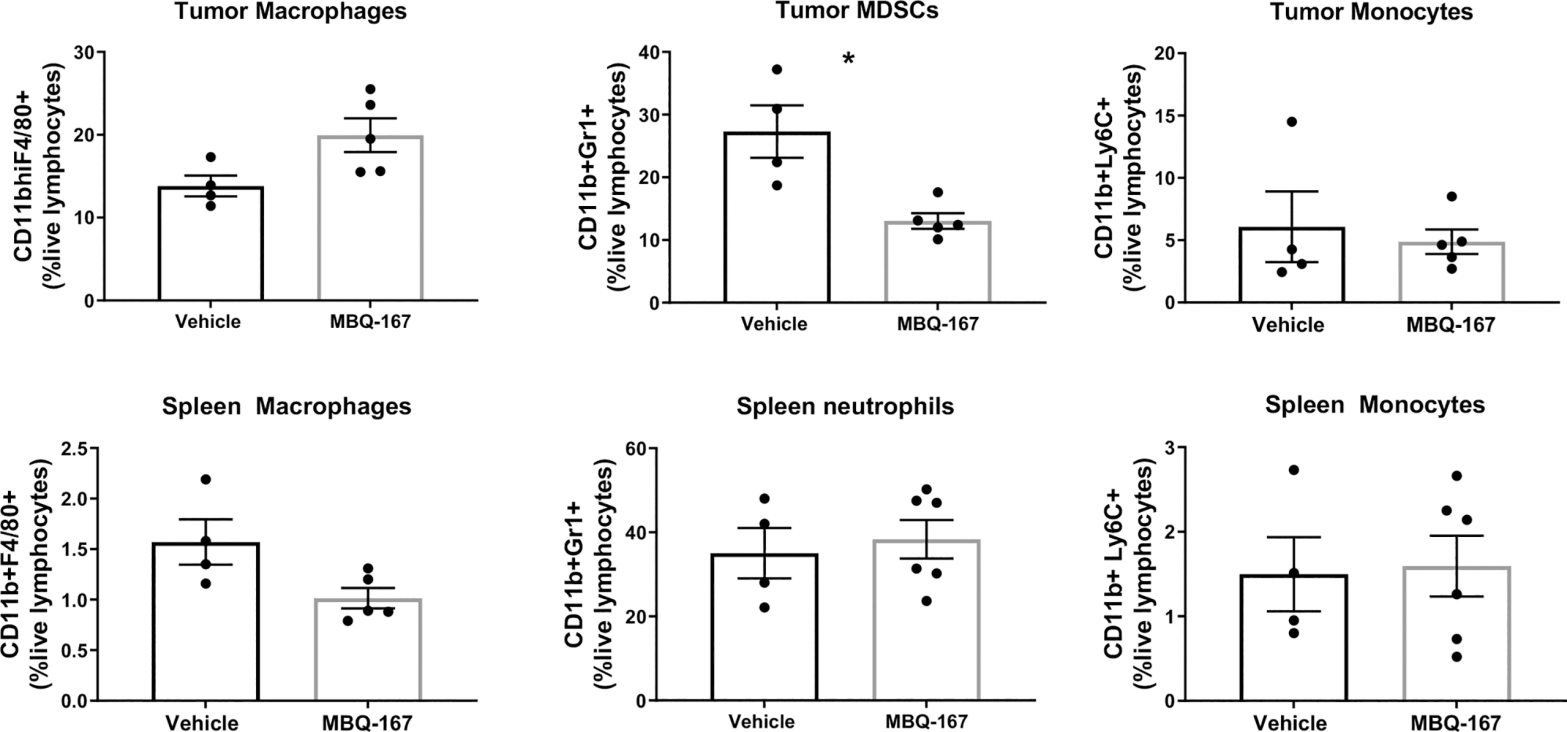
Effect of MBQ-167 on myeloid cells in mouse models. As published in (30), immunocompromised SCID mice were inoculated with GFP-HER2-BM cells, when the tumors reached 100 mm^3^, mice were treated 5X a week with 0 or 5 mg/kg MBQ-167 by IP for 55 days. At necropsy, tumors and spleens were extracted and subjected to flow cytometry using fluorescently tagged antibodies to identify macrophages (CD11b+F4/80+), MDSCs (CD11b+LY6G+), and monocytes (CD11b+Ly6C+) N = 4 – 5, *p<0.05.

The effect of MBQ-167 treatment on myeloid cell activation was also determined from spleens and tumors of imuunocompromised SCID mice implanted with GFP-HER2-BM cells in the mammary fat pad. When the tumors reached 100 mm^3^, mice were treated with 0 or 5 mg/kg BW MBQ-167 by intraperitoneal route 5 times per week. As reported by us, MBQ-167 treatment resulted in a 70% reduction in tumor growth and ~90% reduction in metastasis (30). At the end of the study (55 days), spleens and tumors were extracted and processed to quantify CD11b+F4/80+ macrophages, CD11b+Ly6G+ MDSCs and CD11b+Ly6C+ monocytes by flow cytometry using the gating strategy depicted on **Supplementary Figure S5**. Results show a ~30% significant decrease in spleen macrophages but not tumor macrophages in response to MBQ-167. When relative M1 (CD206 negative) or M2 (CD206 positive) macrophages were differentiated from spleens of SCID mice following MBQ-167, we did not find a distinction between M1 vs M2 macrophages in response to MBQ-167 treatment (Data not shown). When spleen extracts were analyzed for MDSCs, there was no significant change in CD11b+Gr1+ cells in spleens following MBQ-167 treatment; however, tumor MDSCs were significantly reduced by ~57% in response to MBQ- 167 treatment. In contrast, no differences in monocyte frequencies were observed in tumors or spleen after MBQ-167 (**Figure 5**).

**Figure 6:**
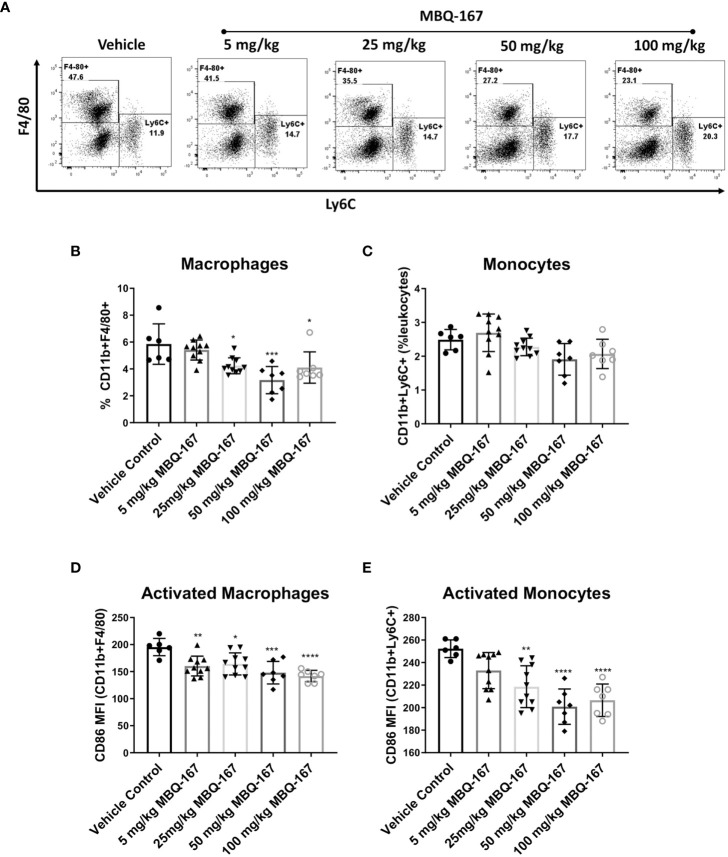
As published in (27), immunocompetent Balb/c mice bearing mammary tumors (~100mm^3^) established from 4T-1 mouse breast cancer cells were treated with vehicle or 0-100 mg/kg MBQ-167 5X a week by oral gavage, for 5 weeks. The spleens were harvested at necropsy for flow cytometry analysis. **(A)** Representative plots for macrophage and monocyte frequencies gated on CD11b+Gr1- cells are shown. The % of macrophages **(B)** and monocytes **(C)** among total live lymphocytes and the expression of CD86 as mean fluorescence intensity on macrophages **(D)** and monocytes **(E)** is depicted (N = 6) *p < 0.05, **p<0.005, ***p<0.0005, ****p<0.00005.

In addition, we implanted immunocompetent Balb/c mice with 4T-1 mouse breast cancer cells and treated them with vehicle, 5 mg/kg, 25 mg/kg, 50 mg/kg or 100 mg/kg of MBQ-167 by oral gavage 5 times per week for 5 weeks. As reported, 50 mg/kg BW MBQ-167 treatment resulted in a significant 60% decrease in tumor growth and a 90% decrease in lung metastases (27). At the end of the study, spleens were collected, and processed to quantify CD11b+F4/80+ macrophages, CD11b+LY6C+ monocytes, and CD86+ activated myeloid cells by flow cytometry. A significant reduction was observed in macrophage and monocyte frequencies in spleens treated with MBQ-167 at concentrations of 25 mg/kg or higher. The most significant reduction was observed in mice treated with 50 mg/kg of MBQ-167 (**Figures 6A–C**). Moreover, the activation of macrophages and monocytes were also significantly reduced after 5 mg/kg and 25 mg/kg of MBQ-167, respectively (**Figures 6D, E**).

### Rac and Cdc42 inhibitors reduce IL-6 secretion in the TME

**Figure 7:**
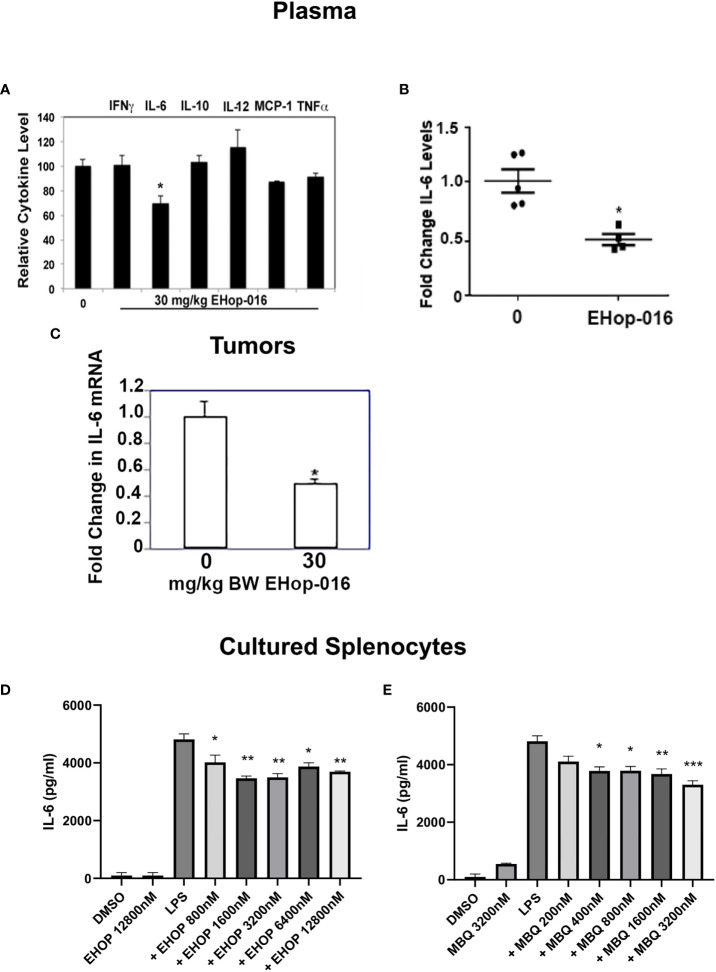
IL-6 is increased in response to Rac/Cdc42 inhibitors. As described in (29), mammary fat pad tumors were established from GFP-HER2-BM cells in athymic nude mice and treated with vehicle or 30 mg/kg BW EHop-016 by i.p. 3X a week for 55 days. At necropsy, plasma and tumors were extracted and subjected to a cytokine array. **(A)** Relative cytokines from plasma following 30 mg/kg BW EHop-016 administration. **(B)** IL-6 levels in mouse plasma following EHop-016 treatment. **(C)** Tumors were extracted and IL-6 mRNA in tumor tissue was measured by qRT-PCR. Fold change in mRNA expression relative to vehicle treated mice is shown. Average fold change ± SEM (N=4) *p<0.05. Splenocytes from SCID mice were cultured with or without LPS (10 µg/mL) and treated with the indicated concentrations of EHop-016 **(D)** or MBQ-167 **(E)**. After 24hrs in culture, supernatants were collected and an ELISA assay was done to quantify IL-6. Statistical significance was calculated using Two-way ANOVA, **p < 0.005, ***p < 0.0005, N.

Macrophages are major producers of pro-inflammatory cytokines, therefore, to determine whether the Rac and Cdc42 inhibitors affect macrophage cytokine production, we quantified the levels of cytokines *in vivo* and *in vitro*. As described in (29), at the end of the 55-day study following 30mg/kg BW EHop-016, administered 3X a week by IP, to nude mice bearing GFP-HER2-BM mammary tumors, the plasma and tumors were extracted and subjected to cytokine arrays. As shown in **Figure 7A**, a cytokine array demonstrated no changes in interferon γ (IFNγ), IL-10, IL-12, Monocyte Chemoattractant Protein (MCP-1), or tumor necrotic factor α (TNFα); but demonstrated a 30% significant decrease in IL-6, as confirmed from an ELISA assay for IL-6 using plasma from the same mice (**Figure 7B**). This decrease was also reflected in IL-6 levels from tumor extracts as quantified by RT-PCR (**Figure 7C**).

We also wanted to determine whether IL-6 production could be affected *in vitro* by Rac and Cdc42 inhibitors using primary myeloid cells. For this, we cultured spleen cells from SCID mice with the TLR4 agonist lipopolysaccharide (LPS), which triggers the production of IL-6. It is important to note that spleens from immunocompromised SCID mice mostly contain macrophages and neutrophils due to the lack of T and B lymphocytes. As expected, LPS increased IL-6 levels in cultured splenocytes, while EHop-016 significantly decreased this spike in LPS-stimulated IL-6 release at concentrations >800 nM (**Figure 7D**). Similarly, when myeloid cells were analyzed by flow cytometry, EHop-016 reduced IL-6 levels by ~50% starting at 800 nM EHop-016 (**Supplementary Figure S6**). A similar significant reduction in LPS-induced IL-6 levels were also observed from cultured splenocytes in response to >400 nM MBQ-167 (**Figure 7E**). The decrease in IL-6 release from LPS-induced splenocytes in response to Rac/Cdc42 inhibitors was relatively modest, compared to the >50% inhibition of IL-6 levels in plasma and tumors from mice treated with EHop-016 for ~2 months. This could be attributed to a cumulative effect of prolonged EHop-016 treatment or additional contribution from cells in the tumor microenvironment, as well as other IL-6 releasing cells such as fibroblasts, in these immunocompromised mice. Taken together, these data implicate Rac and Cdc42 in regulation of immune suppressive myeloid cells, which can be inhibited by the specific inhibitors EHop-016 and MBQ-167.

## Discussion

Rac and Cdc42 are pivotal regulators of both innate and adaptive immune cell migration relevant for immune suppression in the TME (36–38). In hematopoietic cells, Vav1/Rac2 activity is essential for macrophage and neutrophil recruitment *via* adhesion and migration, as well as the phagocytic NADPH oxidase response (21, 39, 40). In addition, differentiation and activation of M2 like macrophages and MDSCs are also regulated by Rac (41, 42). PMA-induced differentiation of monocytes into macrophages activates Rac and Cdc42 without changing their expression (43–45).

The Rac and Cdc42 inhibitors, EHop-016 and MBQ-167, are potent inhibitors of cancer cell migration and metastasis, as our group has demonstrated in different cancer cell types (25– 27, 29, 30, 32, 46–48). However, the effect of these inhibitors on tumor metastasis may not only be caused by direct effects on cancer cells but also through their modulation of immune cells. Herein, we show that similar to their effects on Rac and Cdc42 activation in breast cancer cells, in macrophage-like cell lines, the Vav/Rac inhibitor EHop-016 inhibits Rac activation, and the dual inhibitor MBQ- 167 inhibits both Rac and Cdc42 activation, albeit at ~ 2X higher concentrations than their effective concentrations in cancer cells. Since we have shown MBQ-167 to be absorbed more readily into tumor tissue and to be sustained at longer hours (~8hrs) compared to the ~4hr half-life in plasma (27, 28, 49), we expect the Rac/Cdc42 inhibitors to be more effective in the TME, by affecting cancer and macrophage cell signaling, which results in decreased metastasis.

We also report that macrophage migration is reduced by Rac/Cdc42 inhibitors, without a significant effect on macrophage cell viability at the effective concentrations of 2000 nM for EHop- 016 and 500 nM for MBQ-167. This reduction in migration is paralleled by decreased PAK activity and impairment in actin cytoskeleton structures, similar to the effects observed on cancer cells by EHop-016 and MBQ-167 (25–27, 29). However, higher doses than the ones used for cancer cells were required to reduce macrophage migration *in vitro*. This suggests that different cell types have different sensitivities towards these inhibitors, which may be explained by differential expression levels of Rac and Cdc42, as shown in **Supplementary Figure 3** where equal protein concentrations (25mg) from macrophage or breast cancer lysates with equal actin staining show double the concentration of Cdc42 in macrophages, compared to triple negative breast cancer (TNBC) cells. This may also be an isoform dependent effect because cancer cells express Rac1 and Rac3, while hematopoietic cells express Rac2. In a mouse model of HER2++ breast cancer (29), EHop-016 administration reduced macrophage numbers into tumors as well as metastasis. Since leukocytes are known to promote metastasis *via* cross-talk signaling with cancer cells (7, 24), the observation of reduced macrophage and neutrophil counts in mammary tumors following Rac inhibition validates a dual role for Rac/Cdc42 inhibitors in tumor malignancy. The reduced numbers of macrophages in the tumor may not only be due to impaired migration but to decreased macrophage differentiation since Rac and Cdc42 become activated during monocyte-macrophage differentiation; further studies are needed to demonstrate this (43).

Immune cells such as macrophages, neutrophils, and MDSCs, can be immunosuppressive in the TME and promote tumor cell invasion of the surrounding tissue, intravasation and survival in the circulation, as well as tumor cell arrest, extravasation and persistent growth at metastatic sites, and is correlated with poor patient prognosis (7–12). Between the two major classes of macrophages, the alternative or M2 class predominates in malignant tumors. M2 macrophages are defined by their ability to produce cytokines and soluble mediators that promote an immunosuppressive environment, angiogenesis, tissue remodeling and repair (17, 50, 51). Such immune cell-mediated release of cytokines and chemokines all signal *via* Rac and Cdc42 (52). Further investigation of the macrophages from the spleen also demonstrated that the activation state of macrophages and monocytes was significantly decreased, observed by a decrease in CD86 expression, following MBQ-167 treatment. Although we did not determine *in vivo* that this effect was directly through MBQ-167 on macrophages, it is known that the co-stimulatory molecule CD86 may be induced after TLR engagement, which signals through Rac/Cdc42 pathway. Thus, MBQ-167 may be affecting myeloid cell activation *via* TLR4 signaling (19). Therefore, targeting Rac and Cdc42 in tumor-associated macrophages (TAMs) could inhibit a cascade of events that promote a tumorigenic microenvironment. Since Rac and Cdc42-regulated MDSCs are known to exert immunosuppressive effects in the TME (41, 42, 53), we quantitated the MDSCs in SCID mice bearing HER2++ breast tumors following MBQ-167 treatment. Results show a significant 60% decrease in MDSCs from mammary tumors of mice that received MBQ-167, which indicates an immunoprotective role for Rac/Cdc42 inhibitors. Although this was not the focus of this manuscript, the effect of Rac/Cdc42 inhibitors on intratumoral T cell function is highly important to be determined to understand the impact of these inhibitors in not only innate but also adaptive immune cells.

We also found that Rac/Cdcd42 inhibitors may promote an anti-tumor microenvironment by inhibiting the secretion of the pro-inflammatory cytokine IL-6, which is mostly produced by tumor- associated macrophages. From a cytokine array, we found that IL-6 and not IL-10 or TNFα were significantly changed by Rac inhibition. This indicates a specific effect on IL-6 release, probably through NfκB signaling (20), to promote inflammatory mechanisms. Notably, IL-6 has been found to promote tumor cell proliferation, survival, angiogenesis, and escape from immune surveillance (54–56). IL-6 has been implicated in the dissemination of cancer cells leading to metastasis since it drives cancer cell proliferation and invasiveness while suppressing apoptosis (57). Moreover, IL-6 has been shown to induce the differentiation of M2 macrophages and activation of myeloid-derived suppressor cells (MDSCs) in prostate cancer models (58). Accordingly, we demonstrated that Rac/Cdc42 inhibitors reduced IL-6 secretion *in vivo* after EHop-016 treatment and also in splenocytes following LPS stimulation. Although IL-6 may be produced by macrophages and cancer cells *in vivo*, our results demonstrate that these inhibitors have the potential to reduce inflammatory mediators driving metastasis.

The ability of Rac/Cdc42 inhibitors to decrease pro-inflammatory cytokines implicates them as potential therapeutics for inflammatory conditions. IL-6 is involved in uncontrolled inflammation during autoimmune diseases and chronic inflammatory diseases. For example, IL-6 mostly produced by macrophages in synovial fluid is implicated in rheumatoid arthritis (59). For this reason, blockade of IL-6 is used as a treatment for rheumatoid arthritis and is in clinical trials for other inflammatory conditions, including cancer (60, 61). Therefore, therapeutic intervention with Rac/Cdc42 inhibitors may provide treatment options for inflammatory diseases.

Our results demonstrate the dual role of Rac/Cdc42 inhibitors in inhibiting cancer cell and macrophage migration as well as inflammatory cytokines driving metastasis. Future studies will be aimed in determining the effect of Rac/Cdc42 inhibitors on other immune cell types to better understand their dynamic modulation of the TME. Also, we will determine the effective concentrations for Rac/Cdc42 inhibitors to impair cancer cell and protumorigenic cell migration in the TME, while preserving the function of anti-tumor immune cells.

## Data availability statement

The original contributions presented in the study are included in the article/**Supplementary Material**. Further inquiries can be directed to SD, su.d@upr.edu.

## Ethics statement

The animal study was reviewed and approved by University of Puerto Rico Medical Sciences IACUC.

## Author contributions

AT-S and MR-R designed and performed experiments as a partial requirement of their Ph.D. Dissertations and participated in the preparation of the manuscript. LC-P performed the mouse experiments in **Figure 4** and the subsequent cytokine analysis from mouse tumors and plasma. MM-F supervised the immunohistochemistry studies. SD-E designed, conducted, and supervised experiments, and wrote and edited the manuscript. SD provided funding, advice on experimental design, as well as writing and editing of the manuscript. All authors contributed to the article and approved the submitted version.
