# Supplementary material for: Rac and Cdc42 inhibitors reduce macrophage function in breast cancer preclinical models
Source: Front Oncol. 2023 Jun 16;13:1152458. doi: 10.3389/fonc.2023.1152458 (PMC10313121; doi:10.3389/fonc.2023.1152458)
Supplement: Supplementary file 1 [file DataSheet_1.docx]

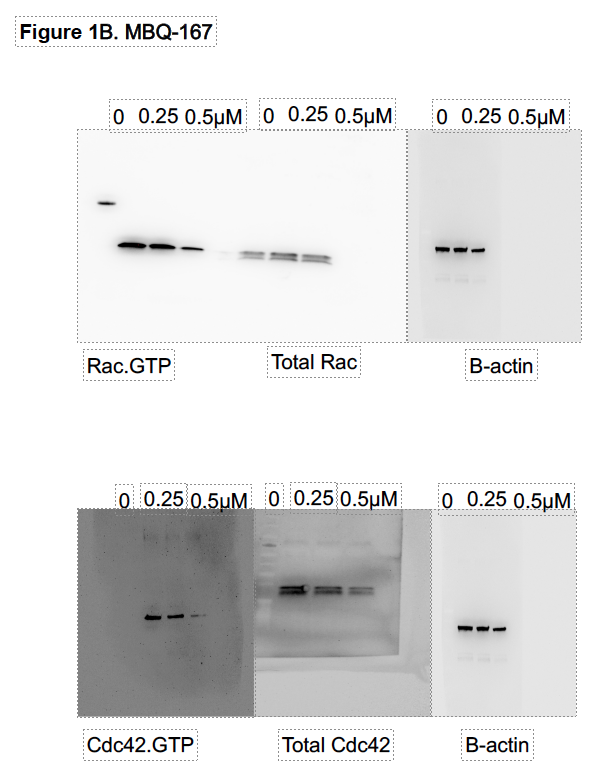
**
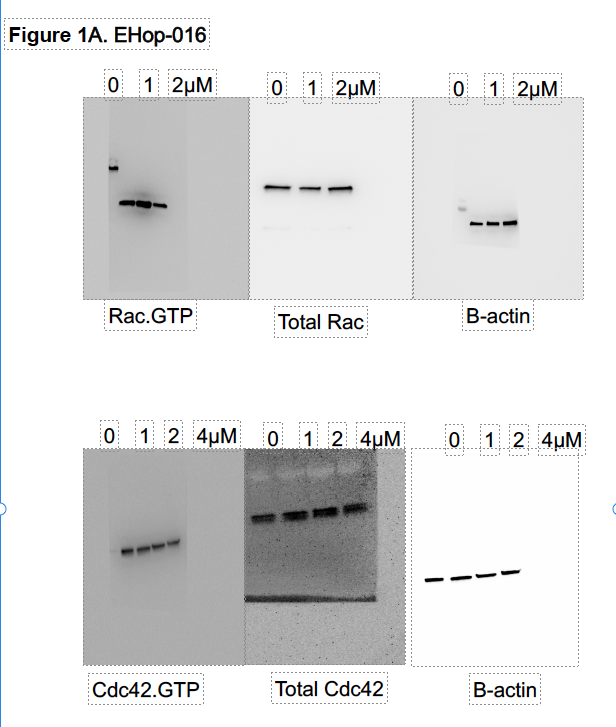
**

**Supplementary Figure S1. Full length western blots from Figure 1.**


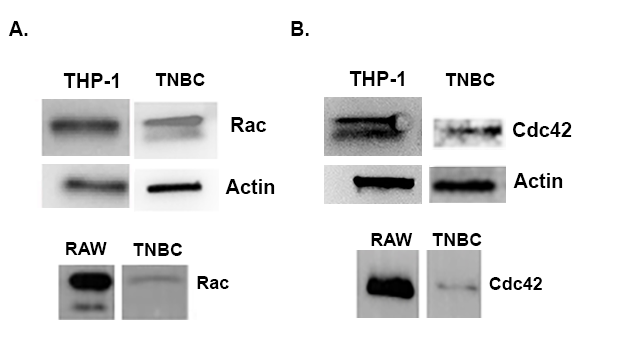


**Supplementary Figure S2. Expression of Rac and Cdc42 in macrophage and cancer cell lines.** THP1 monocytes differentiated into macrophages (top row) or RAW 264.7 rodent macrophage cell line (bottom row) or MDA-MB-231 triple negative breast cancer (TNBC) cells were lysed and equal protein (25 μg) subjected to Western blotting for Rac, Cdc42, or actin. Representative Westerns comparing Rac (**A**) or Cdc42 (**B**) expression at equal actin staining are shown.

**
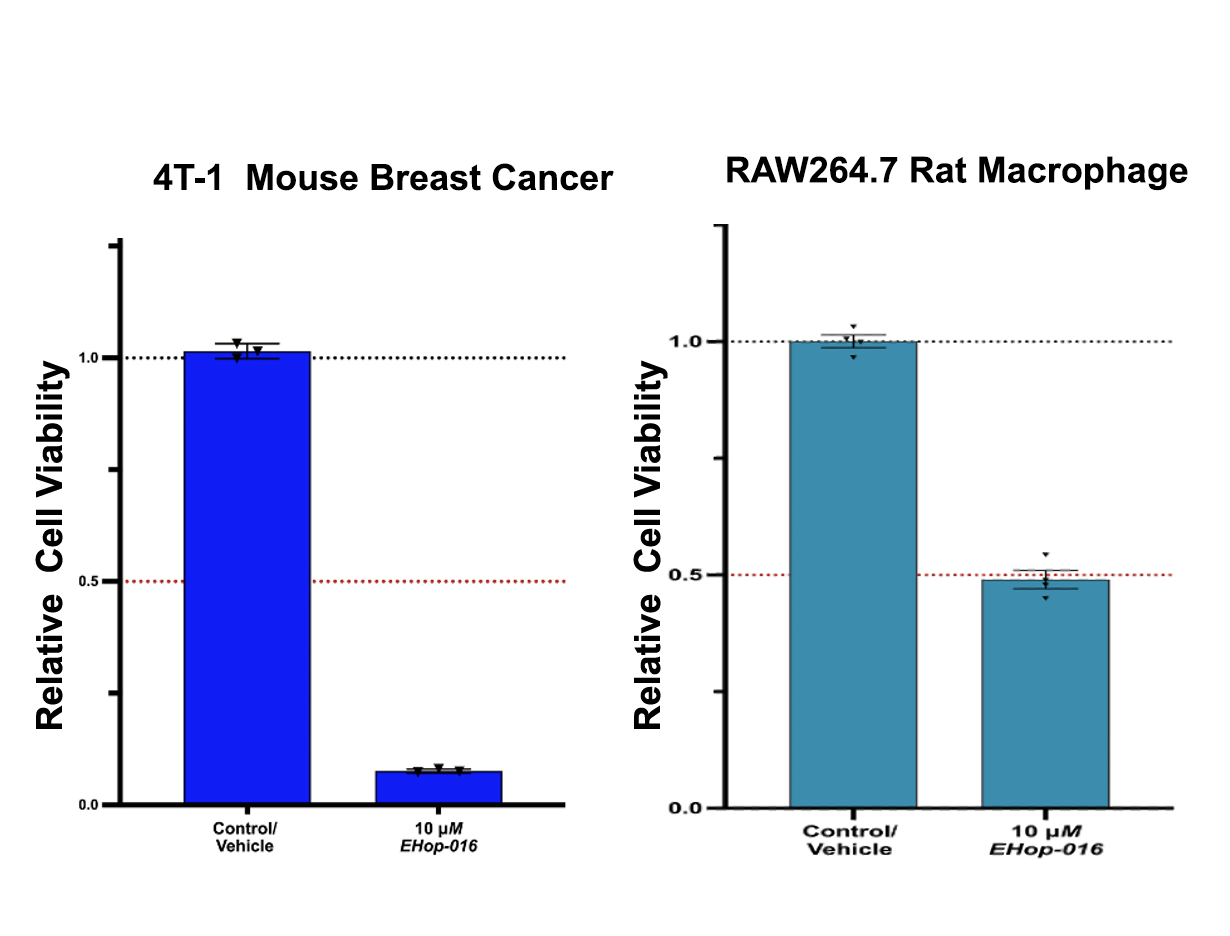
S****upplementary Figure S3**. **Effect of EHop-016 on cell viability**. 4T-1 mouse TNBC cells (left) or RAW 264.7 murine macrophage-like cells (right) were treated with vehicle or 10 μM EHop-016 and subjected to a MTT assay for cell viability. Results shown are N=3.


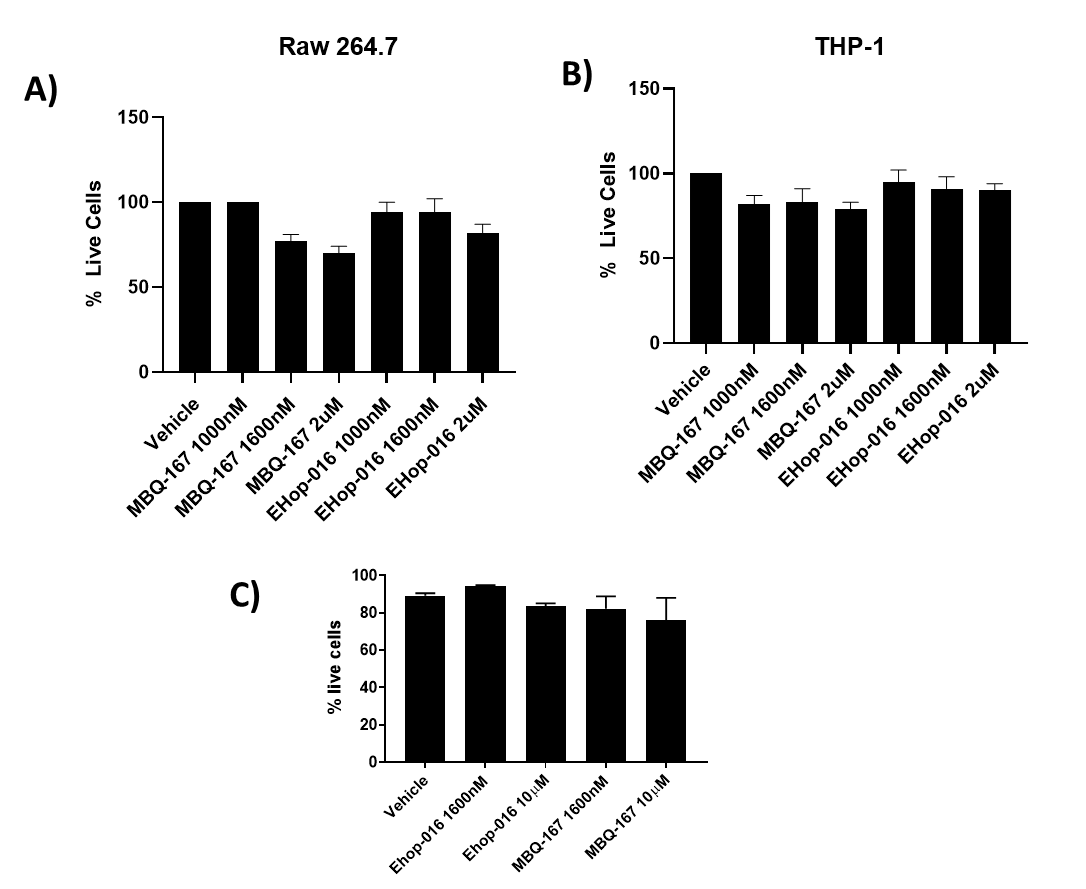


**Supplementary Figure S4**. **Effect of EHop-016 and MBQ-167 on cell viability**. RAW 264.7 murine macrophage-like cells (A) or THP-1 differentiated macrophages (B) were treated with vehicle, or 1 μM – 2 μM MBQ-167 or EHop-016 and subjected to a MTT assay for cell viability. (C) RAW264.7 were cultured and treated with MBQ-167 or EHOP-016 for 24 hrs and the percent of live cells was quantified by flow cytometry by using a live/dead cell marker. Results shown are N=3.

**Supplementary Figure S5.** **Gating strategy for myeloid cell population quantification**.

A representative spleen cell sample analyzed by using FlowJo software is depicted. First, lymphocytes were gated according to FSC and SSC scattering, followed by the exclusion of dead cells (live/dead aqua dye). Then, CD11b+Gr1+ cells were marked as neutrophils, and CD11b+Gr1- were gated for further separation of F4/80 and Ly6C markers. F4/80+Ly6C- cells were identified as macrophages whereas F4/80-Ly6C+ cells were identified as monocytes.

**Supplementary Figure S6. IL-6 expression by myeloid cells after EHop-016 treatment.** Splenocytes from SCID mice were cultured with or without LPS (10 µg/mL) and treated with the indicated concentrations of EHop-016. After 24 hrs in culture, brefeldin A was added to the cultures for 5 hrs. A flow cytometry assay was done to detect IL-6 expression on CD11b+ cells.
